# Development of multidrug-resistant *Escherichia coli* in some Egyptian veterinary farms

**DOI:** 10.14202/vetworld.2022.488-495

**Published:** 2022-02-27

**Authors:** A. A. Samy, Asmaa S. Mansour, Doaa D. Khalaf, Eman A. Khairy

**Affiliations:** Department of Microbiology and Immunology, National Research Centre, Dokki, Cairo, Egypt

**Keywords:** *Escherichia coli*, food of animal origin, misuse of antibiotics, multidrug resistance, poultry

## Abstract

**Background and Aim:**

Food of animal origin is considered a major source of foodborne diseases. In this context, multidrug-resistant (MDR) *Escherichia coli* pose a serious hazard to public health due to the consumption of food contaminated with antibiotics that are used for the treatment of various bacterial infections in farm animals. Therefore, this study aimed to determine the effect of the excessive use of antibiotics on the development of MDR *E. coli* strains in Egyptian poultry, dairy, and meat farms.

**Materials and Methods:**

A total of 1225 samples were randomly collected from poultry, dairy, and meat products intended for human consumption in different governorates. *E. coli* were isolated from the collected samples and subjected to biochemical identification and antibiotic sensitivity tests with antibiotics commonly used in human and veterinary medicine. Then, amoxicillin (AML)- and oxytetracycline (OT)-resistant *E. coli* isolates were subjected to a polymerase chain reaction test to detect the *bla*_TEM_ and *tetA* genes, respectively.

**Results:**

*E. coli* were isolated from 132 out of 350, 148 out of 350, 177 out of 350, and 35 out of 175 poultry, milk, meat, and human samples, respectively. Most of the isolates expressed multidrug resistance, and resistance genes (*bla*_TEM_ and *tetA*) were detected in all the tested AML- and OT-resistant *E. coli* isolates.

**Conclusion:**

Foods of animal origin may represent a source of MDR *E. coli*, which can be a major threat to public health.

## Introduction

Antibiotics are commonly used to treat bacterial infections in humans and animals. However, the association between the extensive use of antimicrobial agents and the progression of resistant bacteria has been well recognized in farm animals [1]. Over the past few years, antibiotic resistance among foodborne pathogens has increased [2]. Antimicrobials are drugs that are used to decrease the growth of microorganisms or even kill them. Antimicrobial resistance arises when bacteria resist these antimicrobial agents. The overuse of such agents to prevent or treat diseases or as growth promoters in animals is considered the main reason for the widespread antimicrobial resistance among farm animals. Antimicrobial resistance genes can be transmitted to humans through the ingestion of the milk or meat products of these animals. Humans and animals are major sources for contamination of other foods and the environment with resistant bacteria [3].

Recently, many researchers have evaluated the antibiotic resistance of bacteria in food products [4,5]. The results showed that a major percentage of the strains isolated from foods of animal origin exhibited antibiotic resistance. In general, resistant microorganisms can transmit their resistance genes to the intestinal flora of humans, which act as a reservoir for these genes [6]. Animals can also act as a source of antimicrobial-resistant pathogens, hence posing a dangerous threat to public health [7]. For example, the high resistance of *Escherichia coli* to ciprofloxacin (CIP) is associated with the use of fluoroquinolones in agriculture [8]. As a result of the genetic flexibility and adaptability of *E. coli* to different environments, these microorganisms have developed numerous mechanisms to resist the action of antibiotics [9]. Such resistance may also be attributed to commensal strains in the lower intestinal tract being repeatedly challenged by antimicrobial pressure during the lifetime of the host. This causes them to acquire many resistance genes and/or develop resistant mutants to survive. Therefore, these microorganisms are considered an indicator of the antimicrobial load on their hosts [10].

This study aimed to investigate the effect of the overuse of antibiotics in Egyptian poultry, dairy, and meat farms on the development of multidrug-resistant (MDR) *E. coli*.

## Materials and Methods

### Ethical approval

This study did not involve any *in vivo* experiments. Poultry, milk and milk products, meat and meat by product samples were collected from the market for microbiological studies, and human samples were obtained from Al-Sharq Laboratory, Haram, Giza, Egypt.

### Study period and location

This study was conducted from June 2018 to May 2020 at the National Research Centre, Cairo, Egypt.

### Samples

A total of 1225 samples were randomly obtained from different Egyptian governorates, including Cairo, Giza, Fayoum, Beni Suef, Menofia, Alexandria, and Qalyubia. All samples were grouped as shown in Table-1.

**Table 1:** Types and numbers of samples collected for examination.

Sample	Types of samples examined	Total
Poultry	Liver, intestinal content, blood, and bone marrow	350
Milk and milk by-products	Yoghurt, raw milk, and Karish cheese	350
Meat and meat by-products	Luncheon, sausages, hotdogs, minced meat, liver, kofta, intestinal contents, and meat	350
Human	Stools obtained from commercial laboratories	175
Total	—	1225

### Isolation and identification of *E. coli* isolates

All samples were individually collected in sterile bags and transferred immediately under aseptic conditions in an icebox at 4°C and transferred to the laboratory for microbiological examination.

Briefly, 1 g or 1 mL of each sample was prepared in normal saline and cultured on the surface of a MacConkey agar medium (Oxoid, UK) to distinguish *E. coli* from other coliforms. The plates were then incubated at 37°C for 24 h. Next, the suspected *E. coli* pink colonies were picked up, streaked on eosin methylene blue (EMB) agar (Lab M, UK), and incubated at 37°C for 24 h. Finally, green metallic shiny colonies were subjected to morphological and biochemical identification, including oxidase, urease, indole production, methyl red, Voges–Proskauer, hydrogen sulfide, and citrate tests along with glucose, lactose, sorbitol, sucrose, and mannitol fermentation [11].

### Antibiotic sensitivity test of *E. coli* isolates

First, the antibiotic sensitivity of the isolates was tested using the disk diffusion technique [12] against the most popular antibiotics used in the veterinary field, including cefradine (CR, 30 μg), CIP (5 μg), oxytetracycline (OT, 30 μg), erythromycin (E, 15 μg), amoxicillin (AML, 10 μg), ampicillin (AMP, 10 μg), and streptomycin (S, 10 μg). Then, *E. coli* isolates were cultured in nutrient broth (Oxoid) and incubated at 37°C for 18-24 h. Next, a bacterial suspension of 0.5 McFarland was prepared and streaked on Mueller-Hinton agar (Oxoid) plates using cotton swabs. Finally, antibiotic disks were placed on the surface of the plates followed by incubation at 37°C for 24 h. After incubation, the inhibition zones were measured (in millimeters) using a ruler.

### Detection of AML- and OT-resistant *E. coli* using polymerase chain reaction (PCR)

A total of 20 AML-resistant and 20 OT-resistant *E. coli* isolates (five isolates from each source) were randomly selected and examined for the presence of β-lactamase AML (*bla*_TEM_) and OT (*tetA*) resistance genes, respectively.

### Bacterial genomic deoxyribonucleic acid (DNA) extraction

DNA extraction from bacterial isolates was performed using the QIAamp DNA Mini Kit (Qiagen GmbH, Germany) as per the manufacturer’s instructions with some modifications. Briefly, 200 μL of the bacterial suspension was mixed with 10 μL of proteinase K and 200 μL of a lysis buffer and incubated for 10 min at 56°C. Then, 200 μL of 100% ethanol was added to the suspension, and the sample was washed and centrifuged. Elution of the nucleic acid was completed using 100 μL of an elution buffer [13].

### PCR amplification

The primers used in this study were purchased from Metabion (Germany) and are shown in Table-2 [14,15]. The reaction was performed in a volume of 25 μL containing 12.5 μL of EmeraldAmp MAX PCR Master Mix (Takara, Japan), 1 μL of each primer (20 pmol/μL), 6 μL of a DNA template, and 4.5 μL of nuclease-free water. Then, the reaction was completed in a Biometra thermal cycler according to the cycling conditions, as shown in Table-3.

**Table 2:** Target genes, primer sequences, amplicon sizes, and cycling conditions.

Target gene	Primer sequence (5′–3′)	Amplified segment (bp)	Reference
*bla*_TEM_	F: ATC AGC AAT AAA CCA GC R: CCC CGA AGA ACG TTT TC	516	[14]
*tetA*	F: GGT TCA CTC GAA CGA CGT CA R: CTG TCC GAC AAG TTG CAT GA	576	[15]

**Table 3:** Cycling conditions for the primers of the *bla*_TEM_ and *tetA* genes.

Gene	Primary denaturation	Amplification (35 cycles)			Final extension

		Secondary denaturation	Annealing	Extension	
*bla*_TEM_	94°C	94°C	54°C	72°C	72°C
	5 min	30 s	40 s	45 s	10 min
*tetA*	94°C	94°C	50°C	72°C	72°C
	5 min	30 s	40 s	45 s	10 min

### Electrophoresis

First, the PCR products were analyzed by electrophoresis using 1.5% agarose gel in 1× Tris-borate-ethylenediaminetetraacetic acid buffer at room temperature (25ºC). To perform a gel analysis, 20 μL of uniplex PCR products were loaded in each gel slot. Then, a 100 bp DNA ladder (Fermentas, Thermo Fisher Scientific, USA) was used to determine the PCR product size. The gel was then visualized by a UV transilluminator, and the data were analyzed with computer software.

### Statistical analysis

Data were computerized and analyzed Statistical Package for the Social Sciences Program Version 13 software for Windows (SPSS Inc., Chicago, III., USA) [16]. Furthermore, significant differences among means were detected by Duncan [17]. One-way classification statistical fixed model was used for statistical analysis as the following:

Y_ij_ = m+G_i_+e_ij_

Where: Y_ij_ is the I^th^ observation of the individual overall means, m is the common mean, G_i_ is the fixed effect of factor, and e_ij_ is experimental error.

## Results

### Prevalence rate of *E. coli* isolates in poultry samples

A total of 132 out of 350 different samples of poultry origin were confirmed to have *E. coli* with a percentage of 37.7%, as shown in Table-4.

**Table 4:** Prevalence rates of *E. coli* isolates from poultry samples.

Sample source	Total no. of examined samples	Recovered isolates of *E. coli*	

		No.	%
Cairo	50	17	34^e^
Giza	50	18	36^d^
Fayoum	50	20	40^b^
Beni Suef	50	19	38^c^
Menofia	50	17	34^e^
Alexandria	50	20	40^b^
Qalyubia	50	21	42^a^
Total	350	132	37.7
p-value		0.000**	

^a,b,c^ Means in the same column bearing different superscripts are significantly different.

** Significant differences at p*=*0.01, *E. coli=Escherichia coli*

### Prevalence rate of *E. coli* isolates in milk and milk by-product samples

For milk and milk by-products, 148 isolates of *E. coli* were obtained from a total of 350 samples, representing a percentage of 42.3%, as shown in Table-5.

**Table 5:** Prevalence rates of *E. coli* isolates from milk and milk by-products.

Sample source	No. of examined samples	No. of recovered isolates of *E. coli*/total no. of original samples	

		No.	%
Cairo	50	19	38.0^d^
Giza	50	22	44.0^c^
Fayoum	50	25	50.0^b^
Beni Suef	50	16	32.0^e^
Menofia	50	22	44.0^c^
Alexandria	50	16	32.0^e^
Qalyubia	50	28	56.0^a^
Total	350	148	42.3
p-value		0.000**	

^a,b,c^ Means in the same column bearing different superscripts are significantly different.

** Significant differences at p*=*0.01. *E. coli=Escherichia coli*

### Prevalence rate of *E. coli* isolates in meat and meat by-product samples

Table-6 shows the incidence rate of *E. coli* among a total of 350 meat and meat by-product samples, among which 177 samples were confirmed to have *E. coli*, representing a percentage of 50.6%.

**Table 6:** Prevalence rates of *E. coli* isolates in meat and meat by-products.

Sample source	No. of examined samples	No. of recovered isolates of *E. coli*/total no. of original samples	

		No.	%
Cairo	50	23	46.0^e^
Giza	50	29	58.0^b^
Fayoum	50	26	52.0^d^
Beni Suef	50	28	56.0^c^
Menofia	50	22	44.0^f^
Alexandria	50	19	38.0^g^
Qalyubia	50	30	60.0^a^
Total	350	177	50.6
p-value		0.000**	

^a,b,c^ Means in the same column bearing different superscripts are significantly different.

** Significant differences at p*=*0.01. *E. coli=Escherichia coli*

### Prevalence rate of *E. coli* isolates in human samples

As shown in Table-7, the recovery rate of *E. coli* from human samples was found to be 20.0% (35 out of 175 samples).

**Table 7:** Prevalence rates of *E. coli* isolates from human samples.

Sample source	No. of examined samples	Recovered isolates of *E. coli*/total no. of original samples	

		No.	%
Cairo	25	6	24.0^c^
Giza	25	7	28.0^b^
Fayoum	25	5	20.0^d^
Beni Suef	25	8	32.0^a^
Menofia	25	4	16.0^e^
Alexandria	25	2	8.0^g^
Qalyubia	25	3	12.0^h^
Total	175	35	20.0
p-value		0.000**	

^a,b,c^ Means in the same column bearing different superscripts are significantly different.

** Significant differences at p*=*0.01. *E. coli=Escherichia coli*

### Antimicrobial sensitivity test of *E. coli* isolated from poultry samples

A total of 132 biochemically confirmed isolates of *E. coli* from poultry samples were tested for their antibiotic profile against seven different antibiotic agents. A pattern of multidrug resistance is observed in Table-8.

**Table 8:** Results of the antibiotic sensitivity test of *E. coli* isolated from poultry samples.

Antibiotic disks	Total no. of *E. coli* isolates	Resistant		Sensitive		Intermediate	
		
		No.	%	No.	%	No.	%
Cefradine	132	70	53.0^f^	44	33.3^a^	18	13.6^c^
Ciprofloxacin	132	67	50.8^g^	33	25.0^b^	32	24.2^b^
Oxytetracycline	132	119	90.2^b^	13	9.8^e^	0	0.0^f^
Erythromycin	132	124	94.0^a^	0	0.0^g^	8	6.0^d^
Amoxicillin	132	110	83.3^c^	22	16.6^d^	0	0.0^f^
Ampicillin	132	100	75.8^d^	27	20.4^c^	5	3.8^e^
Streptomycin	132	93	70.5^e^	4	3.0f	35	26.5^a^
p-value		0.000**		0.000**		0.000**	

^a,b,c^ Means in the same column bearing different superscripts are significantly different.

** Significant differences at p*=*0.01. *E. coli=Escherichia coli*

### Antimicrobial sensitivity test of *E. coli* isolated from milk samples

A total of 148 isolates of *E. coli* from milk and milk by-products were examined for antibiotic resistance and reported in Table-9.

**Table 9:** Results of the antibiotic sensitivity test of *E. coli* isolated from milk and milk by-products.

Antibiotic disks	Total no. of *E. coli* isolates	Resistant		Sensitive		Intermediate	
		
		No.	%	No.	%	No.	%
Cefradine	148	55	37.2^b^	85	57.4^e^	8	5.4^a^
Ciprofloxacin	148	0	0.0^e^	145	98.0^a^	3	2.0^b^
Oxytetracycline	148	30	20.3^c^	111	75.0^d^	7	5.7^a^
Erythromycin	148	63	42.6^a^	85	57.4^e^	0	0.0^c^
Amoxicillin	148	20	13.5^e^	128	86.5^b^	0	0.0^c^
Ampicillin	148	29	19.6^d^	116	78.4^c^	3	2.0^b^
Streptomycin	148	0	0.0e	145	98.0^a^	3	2.0^b^
p-value		0.000**		0.000**		0.000**	

^a,b,c^ Means in the same column bearing different superscripts are significantly different.

** Significant differences at p*=*0.01. *E. coli=Escherichia coli*

### Antimicrobial sensitivity test of *E. coli* isolated from meat samples

As shown in Table-10, an antibiotic sensitivity pattern was observed for *E. coli* isolated from meat and meat by-products, revealing a high incidence of MDR *E. coli*.

**Table 10:** Results of the antibiotic sensitivity test of *E. coli* isolated from meat and meat by-products.

Antibiotic disks	Total no. of *E. coli* isolates	Resistant		Sensitive		Intermediate	
		
		No.	%	No.	%	No.	%
Cefradine	177	75	42.4^b^	95	53.6^d^	7	4.0^f^
Ciprofloxacin	177	0	0.0^f^	174	98.3^a^	3	1.7^g^
Oxytetracycline	177	46	26.0^d^	120	67.8^b^	11	6.2^e^
Erythromycin	177	78	44.0^a^	32	18.1^g^	67	37.9^b^
Amoxicillin	177	46	26.0^d^	102	57.6^c^	29	16.4^d^
Ampicillin	177	55	31.1^c^	86	48.6^e^	36	20.3^c^
Streptomycin	177	11	6.2^e^	85	48.0^f^	81	45.8^a^
p-value		0.000**		0.000**		0.000**	

^a,b,c^ Means in the same column bearing different superscripts are significantly different.

** Significant differences at p*=*0.01. *E. coli=Escherichia coli*

### Antimicrobial sensitivity test of *E. coli* isolated from human samples

Table-11 shows the antibiotic sensitivity profile of *E. coli* isolated from human samples. The results indicate a large number of resistant *E. coli*. The most prevalent resistance was found to be against E in all isolates.

**Table 11:** Results of the antibiotic sensitivity test of *E. coli* isolated from human samples.

Antibiotic disks	Total no. of *E. coli* isolates	Resistant		Sensitive		Intermediate	
		
		No.	%	No.	%	No.	%
Cefradine	35	17	48.5^d^	18	51.5^b^	0	0.0^d^
Ciprofloxacin	35	9	25.7^e^	25	71.4^a^	1	2.9^c^
Oxytetracycline	35	24	68.6^c^	9	25.7^c^	2	5.7^b^
Erythromycin	35	35	100.0^a^	0	0.0^f^	0	0.0^d^
Amoxicillin	35	27	77.1^b^	6	17.1^e^	2	5.7^b^
Ampicillin	35	24	68.6^c^	7	20.0^d^	4	11.4^a^
Streptomycin	35	8	22.9^f^	25	71.4^a^	2	5.7^b^
p-value		0.000**		0.000**		0.000**	

^a,b,c^ Means in the same column bearing different superscripts are significantly different.

** Significant differences at p*=*0.01. *E. coli=Escherichia coli*

### Results of the antibiotic resistance of *E. coli* among all samples

The antibiotic resistance of poultry, milk, meat, and human *E. coli* isolates is shown in Table-12. The most prevalent antibiotic resistance was found to be against E in most samples.

**Table 12:** Results of the antibiotic resistance of *E. coli* from all samples.

Antibiotic disks	*E. coli*/poultry	*E. coli*/milk	*E. coli*/meat	*E. coli*/human
Cefradine	53.0^f^	37.2^b^	42.4^b^	48.5^d^
Ciprofloxacin	50.8^g^	0.0^f^	0.0^f^	25.7^e^
Oxytetracycline	90.2^b^	20.3^c^	26.0^d^	68.6^c^
Erythromycin	94.0^a^	42.6^a^	44.0^a^	100.0^a^
Amoxicillin	83.3^c^	13.5^e^	26.0^d^	77.1^b^
Ampicillin	75.8^d^	19.6^d^	31.1^c^	68.6^c^
Streptomycin	70.5^e^	0.0f	6.2^e^	22.9^e^
p-value	0.000**	0.000**	0.000**	0.000**

^a,b,c^ Means in the same column bearing different superscripts are significantly different.

** Significant differences at p*=*0.01. *E. coli=Escherichia coli*

### Incidence of AML- and OT-resistant *E. coli* in different samples

Table-13 shows the incidence rate of AML- and OT-resistant *E. coli* from different sources. The highest resistance against AML and OT was found among poultry samples with percentages of 83.3% and 90.2%, respectively.

**Table 13:** Prevalence rates of amoxicillin- and oxytetracycline-resistant *E. coli* from different sources.

Sample source	No. of examined samples	No. of recovered *E. coli*		Amoxicillin resistant		Oxytetracycline resistant	
		
		No.	%	No.	%	No.	%
Poultry	350	132	37.7^c^	110	83.3^a^	119	90.2^a^
Milk and its by-products	350	148	42.3^b^	20	13.5^d^	30	20.3^d^
Meat and its by-products	350	177	50.6^a^	46	26.0^c^	46	26.0^c^
Human	175	35	20.0^d^	27	77.1^b^	24	68.6^b^
Total	1,225	492	40.2	203	41.3	219	44.5
p-value		0.000**		0.000**		0.000**	

^a,b,c^ Means in the same column bearing different superscripts are significantly different.

**Significant differences at p*=*0.01. *E. coli=Escherichia coli*

### Detection of AML- and OT-resistant *E. coli* by PCR

**Figure-1:**
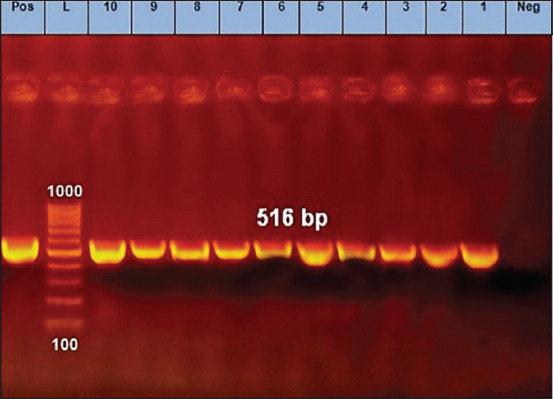
Polymerase chain reaction results of amoxicillin-resistant *Escherichia coli* isolates (*bla*_TEM_ gene). Lane=Lanes 1 to 10 depict positive amplification of the *bla*_TEM_ gene at 516 pb, Neg=Negative control, Pos=Positive control, L=Marker.

**Figure-2:**
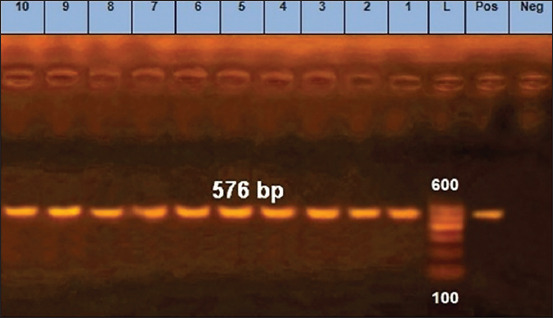
Polymerase chain reaction results of oxytetracycline-resistant *Escherichia coli* isolates (*tetA* (A) gene). Lane=Lanes 1 to 10 depict positive amplification of the *tetA* (A) gene at 576 bp, Neg=Negative control, Pos=Positive control, L=Marker.

As shown in Figures-1 and 2, all the tested AML**-** and OT-resistant isolates were found to carry the *bla*_TEM_ and *tetA* genes, respectively.

## Discussion

All the isolated colonies were Gram-negative, motile, un sporulated bacilli. They demonstrated a lactose fermentation activity on MacConkey agar medium and had a greenish metallic shine on EMB agar. The isolates were also oxidase and urease negative, but indole and methyl red positive. They did not produce acetyl methyl carbinol during the Voges–Proskauer test or hydrogen sulfide on a triple sugar iron medium. They also failed to utilize citrate on the Simmons citrate medium, and they fermented glucose, lactose, sorbitol, sucrose, and mannitol [18].

Overall, 132 out of 350 poultry samples were confirmed to have *E. coli*, amounting to a percentage of 37.7%. These *E. coli* were isolated from the internal organs of chickens, with a prevalence rate of 53.4% [19]. Moreover, 216 *E. coli* isolates were recovered from 270 whole chicken carcass samples [20]. However, the prevalence of colibacillosis was found to be 0.84% in broiler chickens and 0.80% in layer chickens [21]. For milk and milk by-products, 148 isolates of *E. coli* were obtained from a total of 350 samples, amounting to a percentage of 42.3%. Moreover, *E. coli* were reported in milk samples [22], and a high occurrence rate of *E. coli* O157:H7 (34.3%) was found in dairy products [23]. In addition, 222 *E. coli* isolates were obtained from 187 samples of raw milk and dairy products [24], although a lower prevalence rate has been detected in Parseelan *et al*. [25]. Moreover, 177 out of 350 meat and meat by-product samples were confirmed to have *E. coli* (representing a percentage of 50.6%). These results are in agreement with other studies [26,27], although Shaltout found lower prevalence rates for *E. coli* [28]. Finally, the recovery rate of *E. coli* from human samples was found to be 20.0% (35 out of 175 samples), demonstrating a high prevalence rate in humans according to many studies [29-31].

A total of 132 biochemically confirmed isolates of *E. coli* from poultry samples were tested for their antibiotic profile against seven different antibiotic agents. The most prevalent type of resistance was against E, which was detected in 124 isolates (94.0%). Moreover, a high level of resistance was found against other antibiotics, such as OT, AML, AMP, S, CR, and CIP. Notably, many authors have studied the multidrug resistance of *E. coli* in poultry samples and the wide distribution of antibiotic-resistant bacteria in raw chicken meat [32]. With the disk diffusion method, the results showed that nearly 192 (89%) of the 216 isolates were resistant to at least one antibiotic [20].

Moreover, 148 isolates of *E. coli* from milk and milk by-products were screened for antibiotic resistance. The results revealed a high incidence rate of antibiotic resistance against E, followed by CR, OT, AMP, and AML. However, none of the isolates demonstrated resistance to CIP or S. In many other studies, *E. coli* strains isolated from milk and dairy products revealed high antibiotic resistance against different antibiotics [33-36]. In addition, an antibiotic sensitivity pattern was observed for *E. coli* isolates from meat and meat by-products, revealing a high rate of resistance against E, followed by CR, AMP, OT, AML, and S. However, none of the isolates was resistant to CIP. Moreover, 46 *E. coli* isolates from 200 meat samples were identified as extended-spectrum β-lactamase-producing *E. coli* (ESBL-Ec) [37], and other studies revealed high antibiotic resistance [38-41]. Finally, the antibiotic sensitivity profile of human *E. coli* isolates was investigated, and a large number of resistant *E. coli* were detected, mainly to E in all isolates, with a percentage of 100%, followed by AML, OT, AMP, and CR. Many researchers have detected antibiotic resistance in *E. coli* strains isolated from human samples [12,42]. Overall, antibiotic resistance was found in poultry, milk, meat, and human isolates, with the most prevalent rate of resistance being against E in most samples. A high incidence rate of resistance between human isolates and isolates of food of animal origin was also observed. Hence, a special correlation was found in E resistance in human (100.0%), poultry (94.0%), meat (44.0%), and milk (42.6%) samples. In addition, a high incidence rate of antibiotic resistance against AML was found between human (77.1%) and poultry (83.3%) samples. The same correlation was also noticed in the high resistance against OT and AMP between human (68.6% each) and poultry (90.2% and 75.8%, respectively) samples. These results highlight the heavy use of these antibiotics in the poultry farming industry in Egypt, which may point to the transmission of resistance genes from foods of animal origin to humans. A recent study revealed ESBL genes in ESBL-Ec isolated from farmers and their chickens, and genomic similarity analysis demonstrated the involvement of ESBL-Ec between farmers and chickens [43]. These results are in line with other studies [32,34,37]. When PCR was used, all of the tested AML- and OT-resistant isolates were found to carry the *bla*_TEM_ and *tetA* genes, respectively. The same genes were detected in many recent studies on AML- and OT-resistant *E. coli* [9,44-47]. Hence, it can be concluded that there is a direct relationship between the use of antibiotics in poultry farms and the presence of antibiotic-resistant bacteria in humans [48,49]. This highlights the importance of decreasing the use of antibiotics in human and veterinary medicine.

## Conclusion

In this study, *E. coli* were isolated from food of animal origin, including poultry, milk and by-products, and meat and by-products in addition to human stools with high percentages. The isolates were identified with morphological and biochemical techniques. Molecular analysis was not performed for the identification of strains which can be considered as the limitation of the study. It was found that *E. coli* strains were resistant to CR, OT, E, AML, AMP, and S. CIP resistance was found in bacterial isolates concerning poultry and human samples only. This points to excessive use of this antibiotic in poultry farms. Some AML- and OT-resistant strains were confirmed using PCR and all of them were found to have *bla*_TEM_ and *tetA* genes, respectively. These findings indicate a strong relationship between the misuse of antibiotics in the veterinary field and the development of resistant pathogens in humans. Therefore, strict measures should be applied to control the spread of resistant bacterial strains through bacterial culturing, phenotypic identification, and antibiotic sensitivity tests to determine the drug of choice and misuse of antibiotics could be prevented. Moreover, antibiotics used as growth promoters should be avoided in veterinary farms. The government should make great efforts including the use of antibiotics in the poultry and animal farms after consulting veterinarians, encouraging one health surveillance efforts, development of rapid diagnostic tests for antibiotic resistance, and encouraging local and international collaboration for antibiotic resistance prevention and control.

## Authors’ Contributions

AAS: Designed the study and drafted the manuscript. ASM: Revised the manuscript. AAS, ASM, DDK, and EAK: Contributed to the laboratory work. All authors have read and approved the final manuscript.
